# Real-Time Monitoring and Analysis of Zebrafish Electrocardiogram with Anomaly Detection

**DOI:** 10.3390/s18010061

**Published:** 2017-12-28

**Authors:** Michael Lenning, Joseph Fortunato, Tai Le, Isaac Clark, Ang Sherpa, Soyeon Yi, Peter Hofsteen, Geethapriya Thamilarasu, Jingchun Yang, Xiaolei Xu, Huy-Dung Han, Tzung K. Hsiai, Hung Cao

**Affiliations:** 1 School of STEM, University of Washington Bothell, Bothell, WA 98011, USA; mikenike88@msn.com (M.L.); jfor@uw.edu (J.F.); taile92@uw.edu (T.L.); kasap1@uw.edu (A.S.); soyeonyi@uw.edu (S.Y.); geetha@uw.edu (G.T.); 2 School of Medicine, University of Washington, Seattle, WA 98109, USA; ihc3@uw.edu (I.C.); hofsteen@uw.edu (P.H.); 3 Department of Biochemistry and Molecular Biology, Division of Cardiovascular Diseases, Mayo Clinic, Rochester, MN 55905, USA; Yang.Jingchun@mayo.edu (J.Y.); xu.xiaolei@mayo.edu (X.X.); 4 School of Electronics and Telecommunications, Hanoi University of Science and Technology, Hanoi, Vietnam; hanhuydung@gmail.com; 5 School of Medicine, University of California Los Angeles, Los Angeles, CA 90073, USA; THsiai@mednet.ucla.edu

**Keywords:** zebrafish, electrocardiogram (ECG), heart diseases, phenotype screening, ECG pattern recognition, real-time monitoring, machine learning

## Abstract

Heart disease is the leading cause of mortality in the U.S. with approximately 610,000 people dying every year. Effective therapies for many cardiac diseases are lacking, largely due to an incomplete understanding of their genetic basis and underlying molecular mechanisms. Zebrafish (*Danio rerio*) are an excellent model system for studying heart disease as they enable a forward genetic approach to tackle this unmet medical need. In recent years, our team has been employing electrocardiogram (ECG) as an efficient tool to study the zebrafish heart along with conventional approaches, such as immunohistochemistry, DNA and protein analyses. We have overcome various challenges in the small size and aquatic environment of zebrafish in order to obtain ECG signals with favorable signal-to-noise ratio (SNR), and high spatial and temporal resolution. In this paper, we highlight our recent efforts in zebrafish ECG acquisition with a cost-effective simplified microelectrode array (MEA) membrane providing multi-channel recording, a novel multi-chamber apparatus for simultaneous screening, and a LabVIEW program to facilitate recording and processing. We also demonstrate the use of machine learning-based programs to recognize specific ECG patterns, yielding promising results with our current limited amount of zebrafish data. Our solutions hold promise to carry out numerous studies of heart diseases, drug screening, stem cell-based therapy validation, and regenerative medicine.

## 1. Introduction

The zebrafish (*Dario rerio*) model system is an important vertebrate experimental model owing to its small size, low-cost for maintenance, short generation time, amenable genetics, and optical transparency. They have been used to elucidate various aspects of gene function that can be directly related to human genetics and diseases due to their highly conserved genome [1]. Zebrafish are also important for studies in diverse disciplines such as: developmental biology, genetics, pharmacology, toxicology, and neurobiology. Zebrafish are also a common model system for understanding human cardiac development, disease, and regeneration [2,3,4]. Unlike humans, zebrafish hearts can fully regenerate after ventricular injuries, thereby providing a tractable model system to study endogenous heart regeneration [5,6]. The zebrafish model also provides a forward genetic approach to reveal the genetic basis underlying molecular mechanisms of numerous heart diseases. These include arrhythmic diseases, which contributed about 350,000 deaths annually in the U.S. alone. However, the underlying genetic factors of heart disease remains poorly understood [7]. Human genetic studies of patient cohorts have linked mutations in genes affecting cardiac ion channel, sarcomere and desmosome to inherited cardiac arrhythmia [8], which have significantly advanced our understanding of cardiac disease as well as advancing potential therapeutic options. Therefore, it is highly desirable to uncover the identity of causative genes as well as key genetic factors. Last but not least, zebrafish are an excellent model for assessing cardiac toxicology following drug treatment or exposures to deleterious environmental factors [9,10]. For example, the cardiovascular system of zebrafish can be utilized to reveal decreases in heartrate related to *human ether-a-go-go gene* (*hERG*) channel blockade, allowing for drug testing and forward and reverse genetic screens [11,12].

Traditionally, visual immunohistochemistry and genomic approaches have been employed to study the roles of signaling pathways in cardiac regeneration at the cellular level in adult zebrafish. Although possessing obvious advantages, those approaches do not elucidate the functional progress (i.e., regeneration and remodeling of the heart) within the same animal in real-time. Furthermore, these methods do not indicate overall cardiac function of the myocardium. To address this, electrocardiogram-based (ECG-based) methods have been developed to monitor homeostasis, regeneration and remodeling of zebrafish hearts, as well as to screen the phenotypes of studied subjects [13,14,15,16]. Nevertheless, most of the existing ECG acquisition systems present shortcomings as (1) only short periods of time can be recorded, resulting in inconsistent results among different fish; (2) the ECG acquisition requires anesthetized animals limiting intrinsic data collection; (3) the ECG is only suitable for manual one-by-one measurement, which is cumbersome when screening a large number of zebrafish; and (4) the T-wave is hardly detected. 

In recent years, our team has been pioneering in the development of various systems for the acquisition of ECG in zebrafish using microelectrode array (MEA) membranes, providing favorable signal-to-noise ratio (SNR) with full features of P waves, QRS complexes and T waves [17,18]. The obtained ECG signals were clearly distinguishable between heart-injured fish and shams [17]; thus enabling a novel tool to elucidate heart regeneration. However, a hurdle within our system is long-term monitoring over repeated experiments with anesthetized animals, rendering it stressful to the fish and inadequate to provide intrinsic ECG signals. Further, the determination of aberrant ECG patterns in fish was carried out manually, making it onerous for real-time long-term screenings of a large quantity of fish. Recently, our team has introduced a flexible waterproof micro-sensor system to uncover circadian rhythms of freely swimming zebrafish targeting drug-screening applications. While this system was superior to previous models, the wire connections were cumbersome and caused irritations that affected the intrinsic recordings and could not be used with multiple fish simultaneously [18].

In this context, our group has been developing the wired and wireless flexible MEA “ECG jacket” devices to be worn by the animal (zebrafish and neonatal mice) for real-time ECG monitoring and assessment [17,19,20,21]. Recently, we also developed a novel apparatus to house and record ECG signals of multiple awake fish simultaneously [19]. These efforts have provided precise and reliable cardio-physiological assessment in animal models; as a result, data can be simultaneously and continuously collected from different subjects. In this paper, we report our recent efforts on real-time zebrafish ECG acquisition and processing in an automated manner as well as our attempt to leverage advanced data science techniques to analyze and interpret ECG data to detect aberrant patterns.

## 2. Designs, Methods and Implementation

### 2.1. ECG Acquisition Devices

#### 2.1.1. Polymer-Based MEA Membranes

(a) Fabrication

**Figure 1:**
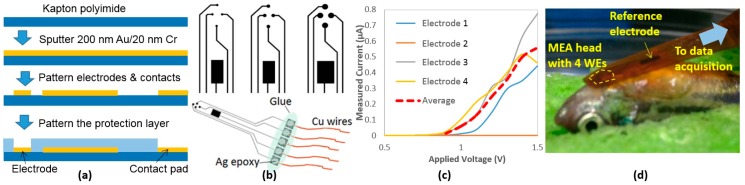
(**a**) Fabrication processes of the MEA membranes; (**b**) Different electrode sizes and the complete device; (**c**) Impedance characterization curves of one 300-µm MEA membrane; (**d**) The MEA on fish.

We have previously developed 4-channel MEA membranes for ECG acquisition in adult zebrafish based on parylene C [17], yielding high spatial and temporal resolution with favorable SNR. Here, we simplified the design and fabricated similar devices on a commercially available polyimide film (125 μm Kapton, Dupont, Wilmington, DE, USA). These newly developed MEA membranes are cost-effective and suitable for acute measurements with anesthetized animals, providing high SNR and spatial resolution. The MEA membranes were fabricated by patterning sputtered metals (200 nm Au on 20 nm Cr) by wet etching followed by an encapsulation process by a 1-μm layer of hardened photoresist (S1813, MicroChem, Westborough, MA, USA), leaving only the electrodes and contacts exposed (Figure 1a). The working electrodes (WE) were designed in a circular shape with three sizes (200, 300, and 500 µm in diameter) while the reference electrode (RE) was designed much larger to maintain proper electrode-tissue interface. Silver epoxy was used to form electrical connections with thin Cu wires and then all contacts were protected by glue using a glue gun (Figure 1b).

(b) Impedance Characterization

Prior to experiments, in order to validate connectivity and characterize the performance of the fabricated electrodes, each MEA membrane was immersed in a saline solution while applying a range of voltages between 0 V and 1.5 V across the WEs and RE. The resulting currents were then measured. The characterization result of a 300-µm MEA membrane is plotted in Figure 1c.

(c) Experiment

All experiments were in compliance with the Institutional Animal Care and Use Committee (IACUC) protocols (#4389-01) approved through the University of Washington to minimize the stress to animals. Adult zebrafish (>6 months) were anesthetized in a buffer solution with 150–200 mg/L tricaine methane sulfonate (Tricaine) and placed on a damp sponge with the ventral side up and visualized under a stereomicroscope [17]. A 2-mm-long horizontal incision was created 0.5 mm caudally to the heart, and then the MEA membrane was located onto the incision placing the four WEs close to the heart and the RE onto the fish body (Figure 1d). Based on our past experience, the MEA did not need to be inside in order to record ECG signals. Once an incision was made, though the wound was recovered, the MEA would be able to measure the signals for weeks. This finding helped ensure that we would not need to perform additional open-chest surgeries from time to time, which possibly cause infection and irritation during the investigation period. 

The four electrodes were connected to 4 channels of a high-gain differential amplifier (A-M Systems, Sequim, WA, USA) and an in-house LabVIEW (National Instruments, Austin, TX, USA) program was used to display, analyze and record the data. The gain was set at 10,000 with filters including a bandpass of 0.1–500 Hz and a notch at 60 Hz to eliminate the unwanted interferences. All signals were then fed into a data acquisition device (DAQ 6000, National Instruments, Austin, TX, USA), being digitized at a sampling rate of 1000 Hz.

#### 2.1.2. PDMS Housing

(a) Fabrication

**Figure 2:**
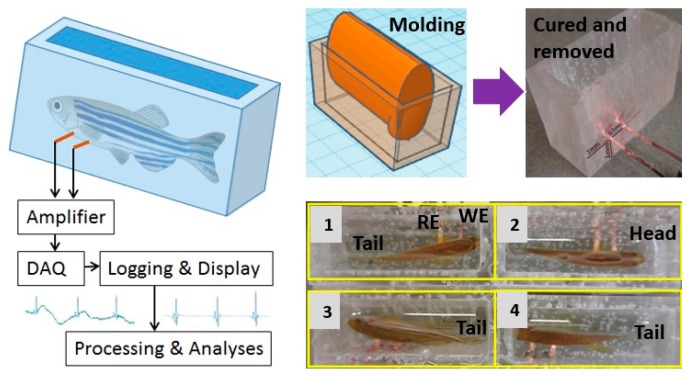
Apparatus formation and the measurement setup.

The in-house apparatus included a multiple-chamber housing made of polydimethylsiloxane (PDMS) using 3D-printed molds with embedded flexible electrodes. The molds were designed using the online-available software TinkerCAD (Autodesk, Inc., Mill Valley, CA, USA) and then formed using a 3D printer. We used two 3D-printed parts, a rectangular tank with an inner length of 53 mm and inner width of 25 mm, and an oval-shape object with a length of 44 mm and a maximum width of 14.7 mm (Figure 2, right panel). First, PDMS (Sylgard 184, Dow Corning, Midland, MI, USA) was cast into the rectangular object and then the oval mold was positioned to form the tapered housing. After PDMS was cured at room temperature in 24 h, the molds were gently removed to make sure the apparatus remained the desired shape. To form the recording electrodes, two strips of 125 μm-thick polyimide (Kapton, DuPont, Wilmington, DE, USA) with sputtered metals were inserted from the side of the apparatus through two thin-cut slits, which were then sealed by applying PDMS. The electrode strips were positioned so that when the fish was loaded into the apparatus, the two electrodes would be securely in contact with the chest and abdominal areas, acting as recording and reference electrodes, respectively. Multiple apparatuses can be used simultaneously for measurement. Figure 2 (lower right panel) demonstrates a setup of four awake zebrafish ready for ECG recording.

(b) Experiment

After the open-chest surgery allowing for good recording of ECG, fish could be loaded into the chambers filled with system water for measurement. In order to maintain comparable SNR of the recorded signal, the water level was lowered so that the fish body would make constant contacts with the electrodes. The pair of electrodes in each chamber was connected to the high-gain differential amplifiers with settings described above.

### 2.2. Noise Attenuation

**Figure 3:**
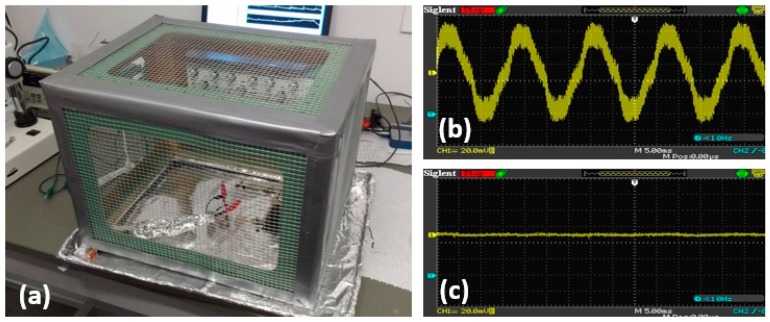
(**a**) The in-house Faraday cage; (**b**,**c**) Ambient EMI without and with Faraday cage.

To reduce ambient electromagnetic interference (EMI), the entire setup was enclosed by an in-house Faraday cage (Figure 3a) and aluminum foil wrapped cables could be utilized. A Faraday cage was constructed of galvanized steel mesh with an aluminum foil lined pan acting as the bottom of the cage. When connected to the ground, this cage would help attenuate the 60-Hz interference from the 110-V AC power line. Its effectiveness was tested by measuring the noise level collected from a conductive wire with and without the Faraday cage covering the wire, the results for which are shown in Figure 3b,c, respectively. In addition, the cables from the MEA device to the amplifier were wrapped in aluminum foil grounded to the amplifier chassis. The ambient noise and interferences will be also further taken care of by digital filters and our signal processing schemes, which will be described in the next sections.

### 2.3. Labview Graphical User Interface (GUI) for Signal Acquistion and Processing

**Figure 4:**
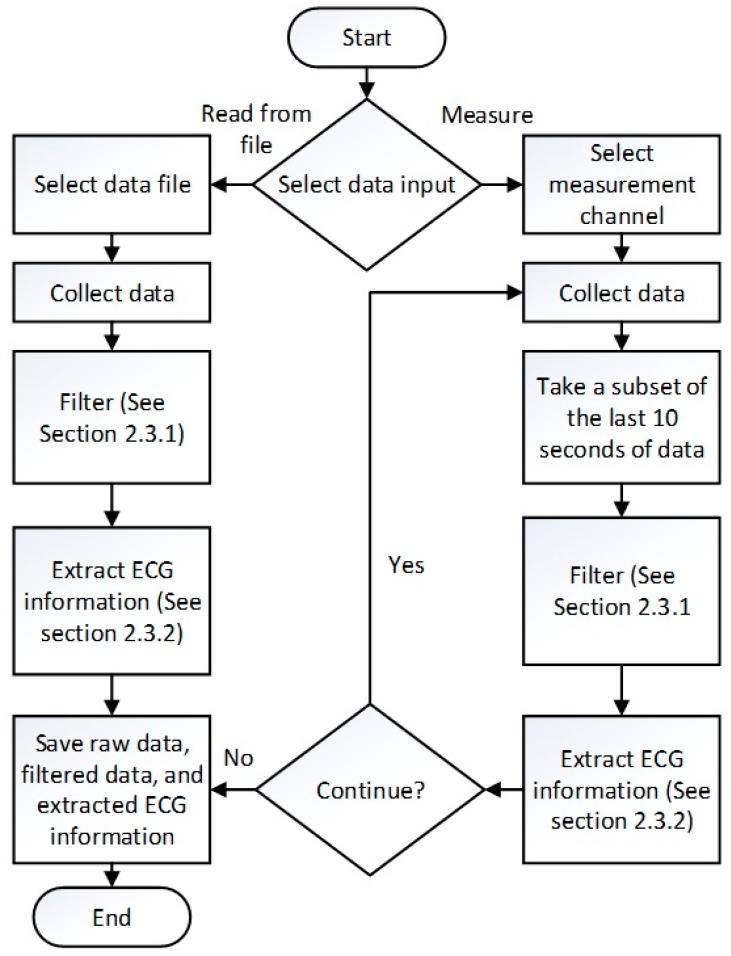
LabVIEW program flowchart.

To collect, process, and analyze the ECG data, a program was created in LabVIEW (National Instruments, Austin, TX, USA). An overview flowchart of the program is showed in Figure 4. Upon starting the program, the user is first presented with the choice to proceed forward using previously acquired ECG data read from a file or measuring live data.

If the choice to read from previously acquired data is selected, the user is then prompted to select file and the location to save the data. The previously collected ECG data is taken from the file, divided into individual channels, and each channel is converted into a waveform. This waveform is sent to a sub-VI that filters out the noise to leave only the relevant portions of the signal (See Section 2.3.1). Then, the filtered waveform is sent another sub-VI that extracts ECG waveform information. The data from the previous sub-VI is taken to a sub-VI that determines whether an anomaly is present in each R-R interval (See Section 2.3.3). Finally, the raw data, filtered data, ECG information for each heartbeat, and a summary document are saved.

If the choice to measure live data is selected, the user is prompted to select the measurement channels and the location to save the data. Once the data recording has begun, the last ten seconds of data is filtered (See Section 2.3.1), the ECG information is extracted (See Section 2.3.2), and the filtered data with the P, Q, R, S, and T wave peaks marked is graphed as data is collected. The extracted ECG information in interpreted by a sub-VI (See Section 2.3.3), which determines whether an anomaly is present. When the experiment is concluded, the user is prompted to press a button indicating that data collection will cease and the collected raw data, filtered data, extracted ECG information for each heartbeat, and a summary document are saved in separate files.

#### 2.3.1. Filtering the Acquired ECG Data

The raw ECG data, collected in real-time or previously, are filtered within a sub-VI. The filtering technique is similar to that described in a National Instruments article on using LabVIEW to process ECG signals [22]. To remove potential high-frequency noise [23], an initial Dolph-Chebyshev lowpass filter was applied. We employed the WA Detrend VI (for wavelet analysis detrending) using the Daubechies6 (db06) wavelet in order to remove the baseline wander [22]. Finally, the remainder of the wideband noise is removed using the Wavelet Denoise VI [22]. 

#### 2.3.2. Extracting ECG Information

The method for extracting ECG information from filtered zebrafish ECG data is based on the windowing algorithm presented in [24]. To extract the peaks of P, Q, R, S and T waves, the sub-VI for feature extraction was designed to look for these peaks within the area between R waves that they are expected to occur in. The R waves are found by using the Peak Detector VI within LabVIEW to find peaks above a threshold of 50% of the maximum amplitude measured. This threshold is low enough that all R waves are detected and high enough that none of the other waves cross the threshold. Then, each R-R interval is divided into a set of “windows” where each wave is detected by the sub-VI. The Q-wave peak and S-wave peak are searched for as the minimums 50 ms before and after the R-wave, respectively. The T-wave peak is the highest peak 15% to 55% of the R-R interval from the first R-wave in the interval. Finally, the P wave is the highest peak 65% to 95% of the R-R interval from the first R-wave in the interval. The locations and amplitudes of the peaks in each R-R interval are collected for each R-R interval. 

In addition to finding the ECG components, the time and amplitude differences between various ECG components are also calculated as outputs of the sub-VI. The calculated time intervals collected are as follows: R-R interval, P-Q interval, Q-R interval, P-R interval, R-S interval, S-T interval, R-T interval, and T-P interval. As the longitudinal intervals are more desirable for ECG signals, the only amplitude information collected is the difference between the R-wave amplitude and the T-wave amplitude. Additionally, the data in the S-T interval are divided into values above and below the isoelectric line then the following information is also calculated as part of the sub-VI for the data points above and below the isoelectric line: The amount of data points, the average value of the data points, and the standard deviation of the data points. The data from this sub-VI is then taken by a sub-VI, which detects the presence of ECG anomalies by comparing the data to currently accepted medical criteria for diagnosing them (See Section 2.3.3).

#### 2.3.3. Detection of ECG Anomalies

The method used for the sub-VI to detect ECG anomalies in the collected zebrafish ECG data was inherited from the diagnosis criteria reported in [25,26]. In our first-generation system, we aimed to diagnose the following ECG anomalies: Sinus bradycardia (A heartrate less than 90 beats per minute (BPM)), sinus tachycardia (A heartrate greater than 150 BPM), sinus arrhythmia (A difference in R-R interval length between the shortest and longest in the last 10 heartbeats of greater than 0.16 s), sinus arrest (A P-P interval greater than 2 R-R interval), ST depression and ST elevation. The numbers chosen here were for zebrafish ECG, based on our past experience and observations. The sub-VI compares the ECG component data collected by the previous sub-VI (See Section 2.3.2) to the presented criteria for each R-R interval. The sub-VI then returns a diagnosis of whether each ECG anomaly is present for each R-R interval. The diagnosis information is then presented on the front panel of the VI, for the latest 10 s of data for the real-time data collection and for the entire data for the previously recorded data. The front panel will display “TRUE” for an anomaly if any of the R-R intervals being presented have been diagnosed with the anomaly by the sub-VI. The indicator for “Normal Rhythm” will only display “TRUE” if no anomalies were detected by the sub-VI for any of the R-R intervals. When the data collection, filtration, analysis, and interpretation have completed, a summary file is created along with the raw data files. 

Along with using user-set criteria for simple ECG anomaly detection on the GUI, the program will be ultimately coded to integrate with an ECG-anomaly library established using machine learning techniques for real-time detection of a wide range of complex anomalies (See Section 2.4).

### 2.4. Machine Learning Approaches

Machine learning (ML) has been employed to great effect in a variety of fields including medicine [27]. ML can be broken down into three main categories: supervised learning, unsupervised learning and semi-supervised learning [28]. Supervised learning relies on human input to supply the learning algorithm with labeled class data, unsupervised learning classifies data samples based on the features inherent within the data, and semi-supervised learning uses a combination of both. Each approach has certain areas in where they excel. Two of the biggest challenges when identifying ECG anomalies in zebrafish, are the high noise level inherent in the signal, and the lack of well-defined diagnostic parameters for specific-disease patterns. This makes more traditional methods of algorithmic identification extremely challenging. Despite this, a trained eye is often capable of identifying abnormal features, even when an algorithm fails. The visual and “fuzzy” nature of this type of identification, makes Convolutional Neural Networks (CNN) and clustering approaches intriguing choices for the identification of cardiac abnormalities. CNN approaches have been applied successfully to similar signal-based analysis with their application to time-frequency spectrograms for voice and emotion recognition [29]. For this reason, in this paper, we investigate the use of CNN and K-means clustering to interpret zebrafish ECG data. We attempted to demonstrate the use of ML for training and recognition of three specific patterns: Atrioventricular Block (AVB), Sinus Arrest (SA) and ST Elevation (STE), appeared in those gene breaking transposon (GBT) mutant lines, GBT422, GBT410, and GBT235, respectively. Each signal in the dataset consists of approximately 4 to 5 beats, which is roughly equivalent to 3000 samples at 1000-Hz sampling frequency. The data was pre-processed and filtered prior to training, the dataset size and training parameters were adjusted as necessary and the results were compared. 

#### 2.4.1. K-Means Clustering

Clustering algorithms are often utilized for unlabeled feature detection and classification. K-means clustering is a simple and straight-forward iterative approach and is one of the most well-known clustering algorithms in use today [30]. K-means has been successfully applied in a variety of fields, including human cardiology, obtaining high accuracy [31]. The high-level noise in the zebrafish ECG poses unique challenges. Moreover, each type of disease patterns has its own unique morphology, making clustering analysis is a good method for classification.

**Figure 5:**
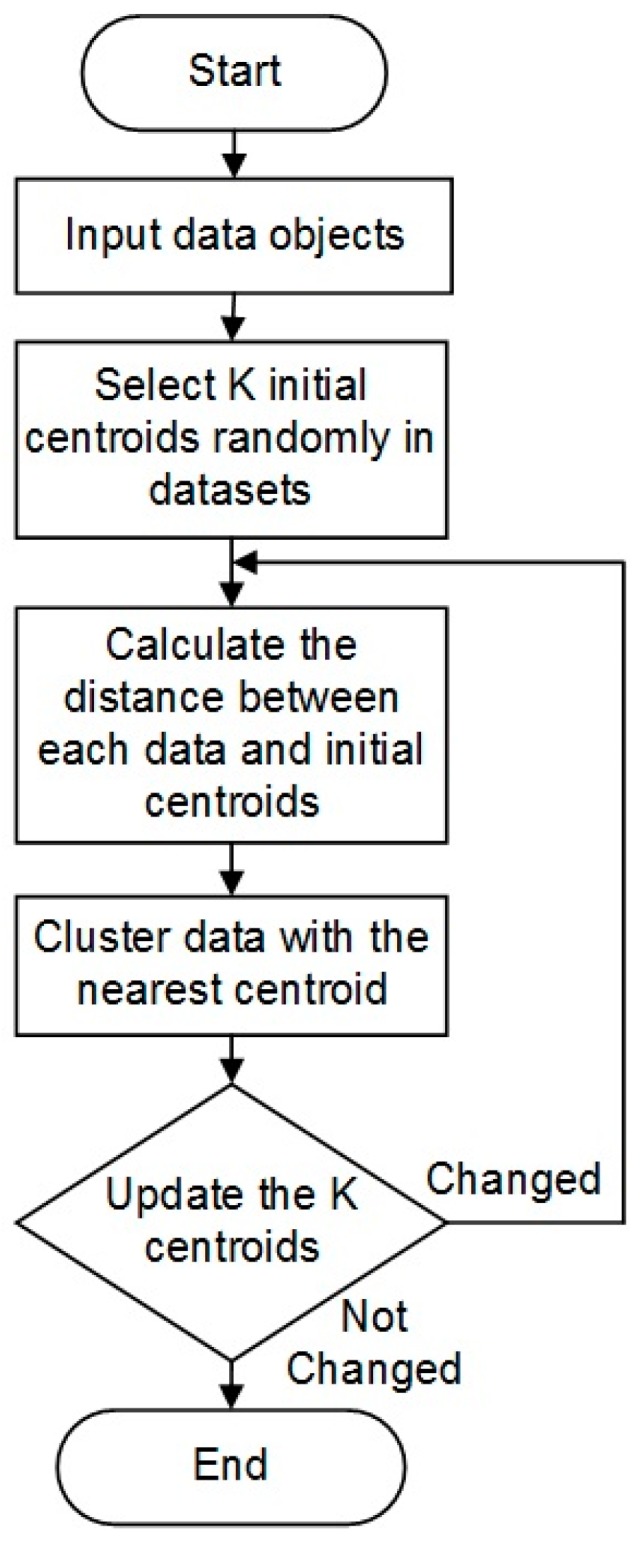
K-means clustering algorithm.

K-means works by partitioning data into k-numbers of clusters with one centroid defined for each cluster. The initial values of the centroids may be randomly selected. For each data point, the distance to each cluster is calculated. If the data point is closest to its initial cluster, the data point is left in that cluster, otherwise it is moved to the closest cluster [32]. This is repeated until a complete pass is made. The details of this approach are illustrated in Figure 5.

#### 2.4.2. Artificial Neural Networks

Researchers in the field of human cardiology trained a Deep Neural Network (DNN) in order to detect premature ventricular contractions, yielding 99.41% accuracy [33]. Convolutional Neural Networks (CNNs), a type of DNN, consistently deliver great performance in image classification tasks. For instance, Google’s Inception v3 is capable of classifying images from 1000 classes with a Top-5 error rate of 3.58% [34]. While neural networks can boast some impressive accuracy, one of their main trade-offs is that training one from scratch generally requires an elephantine amount of labeled data. Google’s Inception v3 model, for example, was trained on 1.2 Million labeled images. Similarly, the neural network used in [33] was trained on over 80,000 samples. Fortunately, it is still possible to get favorable results from an ANN with a smaller dataset by utilizing a technique called “transfer learning” [35]. Transfer learning is the generalization of information learned for one task, to another task. In the context of DNNs, a previously trained neural network can be repurposed to work on a different problem. In order to accomplish this, the classification layer is replaced, and the final few layers are trained on a new dataset. During training, the weights of these final layers are adjusted to reflect the new data. This approach is possible because the output of this penultimate layer is essentially a feature vector, which is then used by the classification layer. If the features are similar enough to the new task, the layer weights only need to be slightly adjusted to fit the new data. This approach is applied here to investigate whether generic visual features from a pre-trained CNN may be applied to successfully bootstrap recognition of cardiac anomalies in small-dataset environments, the last layer of a pre-trained image recognition CNN is retrained utilizing ECG data.

Here, we used Matlab to transform the 1-dimensional ECG signals into 2-dimensional images, resulting in square greyscale plots. The amplitude values of each segment were mapped to greyscale colors. Each segment was then mapped to a square greyscale matrix using a “z-layout”, with the first sample occurring in the upper left hand corner of the matrix and the last sample occurring in the lower right hand corner. The signals were truncated to 2916 sample to accommodate the square layout. The CNN was constructed using Keras (an open source neural network library written in Python) with a Tensorflow backend [36,37]. Google’s Inception v3 model was imported without its final layer and the model was configured with inception’s ImageNet pre-trained layer weights. The following layers were then added to the top of the inception architecture: a 2D global average pooling layer, two densely connected layers with 1024 and 256 parameters both utilizing Rectifier Linear Unit (ReLU) as the activation function, then a final densely connected layer using Softmax activation. The layers on the original Inception model were frozen and the new layer was trained for 50 epochs utilizing only the output features from the original model, with rmsprop utilized as the activation function and Sparse Categorical Cross-Entropy used for the loss function. The layers after the final convolution layer (the final 12 layers) were then unfrozen and the model was retrained to fine-tune the weights on the 12 unfrozen layers. The model was trained for 400 epochs, the loss function employed was sparse categorical cross-entropy, the metric used was accuracy and the optimizer used was stochastic gradient decent with a learning rate of 0.0001 and a Nesterov momentum of 0.9, sparse categorical cross-entropy was used as the loss function.

#### 2.4.3. Algorithm Evaluation

To ensure the validity of these approaches, 10-fold cross validation was employed, where the models were trained from 9 non-overlapping data subsets and tested from the remaining subset, and this process was repeated until all sets have been used for both testing and training and the results were then averaged. Precision, recall and F1 score were used as criteria for classifier evaluation [38]. These parameters are related to True Positives and False Positives (TP/FP), which refer to the number of predicted positives that were correct/incorrect, and similarly for True and False Negative (TN/FN) [38]. Here, TP_AVB_, TP_SA_, TP_STE_ are true positives; FP_AVB_, FP_SA_, FP_STE_ are false positives; FN_AVB_, FN_SA_, FN_STE_ are false negatives; and TN_AVB_, TN_SA_, TN_STE_ are true negatives for 3 classes AVB, SA and STE, respectively. The average accuracy, precision, recall and F1 score are calculated respectively by Equations (1)–(4).

(1) $$\mathrm{Average}\;\mathrm{Accuracy}\;=\;\frac{1}{N}\sum_{i=1}^{N}\frac{{\mathrm{TP}}_{i}{+\mathrm{TN}}_{i}}{{\mathrm{TP}}_{i}{+\mathrm{TN}}_{i}{+\mathrm{FP}}_{i}{+\mathrm{FN}}_{i}}$$

(2) $$\mathrm{Precision}\;=\;\frac{1}{N}\sum_{i=1}^{N}\frac{{\mathrm{TP}}_{i}}{{\mathrm{TP}}_{i}{+\mathrm{FP}}_{i}}$$

(3) $$\mathrm{Recall}\;=\;\frac{1}{N}\sum_{i=1}^{N}\frac{{\mathrm{TP}}_{i}}{{\mathrm{TP}}_{i}{+\mathrm{FN}}_{i}}$$

(4) $$F1\;\mathrm{score}\;=\;\frac{2\;\;\times \;\mathrm{Precision}\;\;\times \;\mathrm{Recall}}{\mathrm{Precision}\;+\;\mathrm{Recall}}$$

where N is number of classes and i (1, 2 and 3) represents AVB, SA and STE, respectively. Results were plotted and confusion matrices were used to indicate the efficacy of each training model. The number of correct and incorrect predictions of testing data will be illustrated in the confusion matrix along with the actual values. The diagonal of the matrix represents the outcomes where the predicted label was equal to the actual label.

## 3. Results

**Figure 6:**
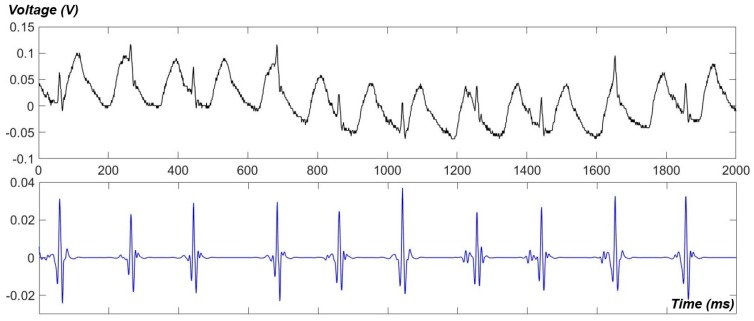
ECG signals, raw (**upper**) and de-noised (**lower**), recorded from an awake fish, using our newly developed PDMS apparatus.

Using our newly developed apparatus, ECG data of awake fish were obtained. A raw signal and its de-noised one are showed in Figure 6. As can be seen, the gill-motion artifacts dominated the raw signal but they were successfully eliminated by our de-noising technique using the Wavelet transform [17]. However, P waves and T waves were hardly detected in awake signals. The measurement with awake fish also revealed that the intrinsic heartrate may be much higher than our past anticipations (90–120 BPM). Heartrates of over 200 BPM were usually found in awake recordings (Figure 6); however, it might be due to the fact that the fish was irritated in a confined and narrow housing.

**Figure 7:**
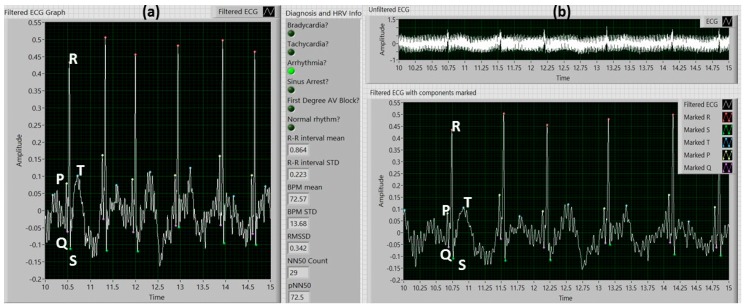
(**a**) Main program front panel with filtered ECG data, marked ECG components, and anomaly diagnosis information; (**b**) Unfiltered and filtered zebrafish ECG data with ECG components identified.

Figure 7a illustrates an example of the front panel of the VI with zebrafish ECG data previously recorded using the MEA membrane, showing the ECG components marked and displaying the diagnosis information along with the following information related to heartrate (HR) and heartrate variability (HRV): The mean BPM for all the R-R intervals, the standard deviation of the BPMs of all the R-R intervals, the mean R-R interval length, the standard deviation of the R-R interval lengths, the square root of the mean of the squares of the differences between adjacent R-R interval lengths (RMSSD), the number of adjacent R-R intervals with a difference greater than 50 ms (NN50 Count), and the percentage of adjacent R-R intervals with a difference greater than 50 ms (pNN50). Arrhythmia was detected, thus the “Arrhythmia” radio button was set to green. 

The front panel of the main VI with a raw ECG signal and its filtered waveform, is showed in Figure 7b. All P waves, QRS complexes and T waves were successfully detected. For this particular signal, the success rates for peak detection was 100% for P, Q, R, S, and T peaks when compared to the peaks detected manually. The noise was reduced from approximately 1 mVpp to 0.05 mVpp, before and after de-noising, respectively. Table 1 displays a summary of the anomalies detected and the R-R intervals in which each was present.

**Figure 8:**
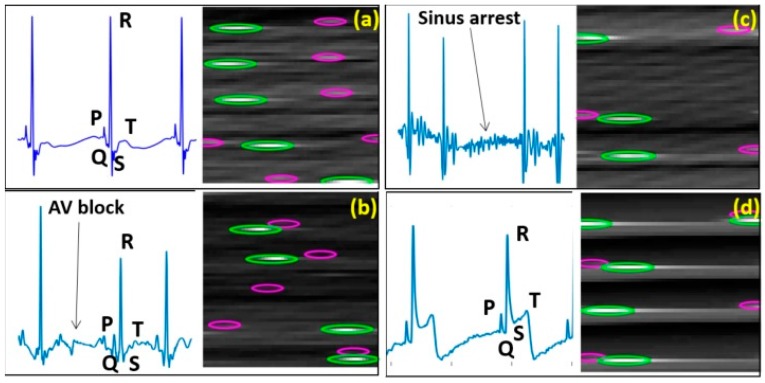
Exemplary standard ECG patterns and exemplary conversion images for CNN training to indicate general differences in the presentation of wave morphology. (**a**) Control fish; (**b**–**d**) Mutant lines with phenotypes of AVB, SA and STE, respectively. Green: R peaks; Pink: P peaks.

The ECG waveforms of control, AVB, SA and STE fish as well as their conversion images prepared for the CNN approach are showed in Figure 8.

**Figure 9:**
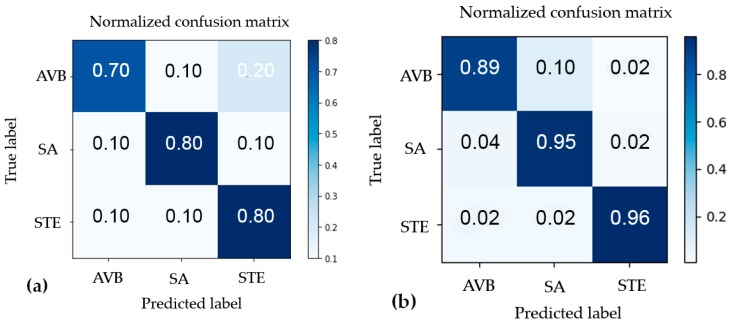
(**a**) The confusion matrix for k-means clustering-based classification; (**b**) The confusion matrix for CNN-based classification.

Figure 9a depicts the results from the K-means clustering algorithm, which had an overall accuracy of 76% while the CNN approach used with 488 samples, obtaining an overall accuracy of 93.26%, are showed in Figure 9b. Both the K-means model and the ANN performed best when distinguishing STE with an accuracy of 80% and 96%, respectively; while it was more challenging to differentiate AVB and SA for both approaches.

Table 2 illustrates evaluation metrics for the two different models, including precision, recall and F1 scores for each cluster.

## 4. Discussion

Both of our acquisition approaches possess valuable novelties to provide a paradigm shift in heart-disease studies and drug testing. The MEA membrane provided multi-site ECG acquisition [17], with favorable spatial resolution as well as SNR, which other systems fail to provide [17]. The wireless feature under development will further enable real-time monitoring of intrinsic ECG with freely swimming fish [20,39]. Although full-feature ECG signals may not be acquired using the newly developed PDMS apparatus, it provides high-throughput screening of multiple fish simultaneously and would be suitable for those applications in which only heartrate and heartrate variability features are of interest. We noticed that if the fish is much smaller than the housing, there may be significant mechanical artifacts that affect the ECG signal quality. Here, we attempted to design our housing in a tapered shape, aiming to resolve this. However, with a large variation in fish size, there would be no ideal housing. This suggested us to design a microfluidic channel-inspired system for ECG screening of multiple embryonic fish, similar our current PDMS housing design and the previous work published by Rendon-Morales et al. [40]. In embryonic stages, fish have similar size, thus we expect to be able to optimize the apparatus. Further, if the expected phenotypes can be observed in embryonic stages, this would help shorten the study time significantly. Noticeably, based on the results we obtained using the current PDMS housing, we speculate the intrinsic heartrate of zebrafish may be in the range of ~200 BPM, providing an invaluable information in establishing new standards and criteria for zebrafish cardiac physiology.

Previously, the process of diagnosing anomalies in zebrafish ECG data required the raw data to be uploaded to a separate program for filtration then the waveform features were extracted manually and beat-by-beat observation was performed to find aberrant patterns. This process was exceedingly time consuming and resource intensive, even for interpreting ECG data from a single subject. In order to facilitate biological investigations, the process of going from raw ECG to detecting anomalies in the signal needs to be expedited to allow for the monitoring of progressively larger groups of test subjects over greater periods of time. The aim of our in-house LabVIEW program is to allow real-time and pre-recorded ECG data to be easily collected, the unwanted noise filtered out, the waveform features extracted, and a diagnosis of select anomalies within the signal to be presented, all in a single program. This will allow scientists to receive diagnosis of ECG anomalies, as well as cardiac information, in real-time while collecting data. Further, raw ECG data obtained by using other means and/or other researchers can be rapidly interpreted by the program to allow for quick assessment and analysis. To ensure the robustness of the program, the GUI has been, and continues to be, tested using fault injection and resolving any issues that such tests have presented. In addition, the program gives constant instructions to the user in order to guide them through using the program and will warn the user when action needs to be taken to correct a problem along with specific details about the nature of the problem that requires correction. Detailed notes on the operation of individual sub-VIs have also been created to aid future modifications to the GUI without potentially damaging the primary functionality of the program.

Unsupervised algorithms such as clustering compensate for a lack of labeled data by their ability to analyze unlabeled datasets and detect patterns that haven’t been discovered before. In contrast, the CNN compensated by utilizing a supervised image-based approach, which made labeling the training set much faster. These methods are thus a great fit to detect zebrafish ECG anomalies due to their capability to deal with less well-defined inputs and a lack of well-defined diagnostic criteria. CNN and K-means clustering approaches share the ability to extract latent features from complex data, which makes both a good choice for this type of determination. Both of the methods employed allowed us to use quick visual evaluation of the signal by a human, allowing for more data to be labeled quickly for training. There are a few likely reasons for the lower accuracy of K-means relative to the CNN (Table 2). First, K-means clustering is known to suffer from problems due to high-dimensional data, especially in cases where data distribution causes the distance between the points to be uniform. This could potentially be addressed by reducing the dimensionality of datasets in the form of feature selection using methods such as principal component analysis (PCA) where irrelevant features or those that show high correlation with other features are removed. Second, it may be due to the randomized selection of initial centroids. The output of representative clusters from K-means is heavily dependent on the selection of the initial centroids. In the traditional K-means approach used here, these were randomly selected. To improve the accuracy, a different clustering algorithm could be used, features could be manually engineered to reduce their dimensionality, or K-means could be modified to allow for controlled selection of the centroids. In contrast to K-means clustering, the utilized CNN method performed beyond expectations given the small amount of labeled data. One aspect of the training that may be the key to the performance of this model, was curtailing the training to 400 epochs, as models trained beyond this point tended toward over-fitting. If more data were used to train the CNN, the training could likely be extended further than 400 epochs with a positive outcome.

The utilization of an image-based method and a pre-trained CNN allowed us to circumvent many of the issues encountered with ECG data from zebrafish. While classification with K-means did not perform as well as the CNN did in this case, unsupervised clustering is an excellent tool for the discovery of common features within datasets. Further, the strengths of the two approaches create an opportunity to increase the performance by combining both methods into a semi-supervised approach, possibly enabling more-efficient evaluation of zebrafish ECGs.

## 5. Future Work and Conclusions

For future improvements, our LabVIEW program has been designed to allow for integration with cloud services using available toolkits. For instance, the LabVIEW toolkit for Amazon web services (AWS) provides developers with interfaces from real time LabVIEW applications to cloud services for storage and data analytics. The LabVIEW Analytics and Machine Learning toolkit in particular will be used to train machine learning models to discover patterns of data and diagnose anomalies. The GUI’s anomaly diagnosis criteria adapted from the available medically accepted definitions continues to be updated. We will also investigate, compare and optimize various machine approaches for determination and interpretation of specific ECG anomalies. In parallel, we will use microinjection and genome editing technology to produce a wide range of heart-disease zebrafish mutant lines to support data collection and then an abnormal-ECG library will be established using the optimized machine learning schemes to support both biological studies in zebrafish and health monitoring in cardiac patients as well as healthy populations.

Impressed with progress in machine learning and artificial intelligence, Elon Musk, the CEO of Tesla, recently even warned about the potential harm of artificial intelligence to future society. However, so far it is mainly in the consumer and entertainment industries where regulations are lax. In the biomedical field, it is still immature. Therefore, our work presented in this paper has paved the way for numerous biological studies by marrying novel acquisition approaches and cutting-edge data science innovations, namely machine learning and deep learning. It also holds the translational implication to enable automated diagnosis, distanced care, e-care and personalized medicine for humans.

## Figures and Tables

**Table 1:** Summary table of anomalies detected in the ECG signal presented in Figure 7a.

R-R Interval Number	Anomaly/Anomalies
1–2	Bradycardia
3–41	Bradycardia, Arrhythmia

**Table 2:** Classifier results.

Abnormality	Precision		Recall		F1	
	K-Means	CNN	K-Means	CNN	K-Means	CNN
AV Block	0.78	0.95	0.70	0.89	0.74	0.92
ST Elevation	0.8	0.98	0.80	0.96	0.80	0.97
SA	0.73	0.86	0.80	0.95	0.76	0.90
Average	0.77	0.94	0.77	0.93	0.77	0.93
